# A spatiotemporal atlas of giant-cell formation during *Meloidogyne incognita* infection in tomato roots

**DOI:** 10.1186/s43897-026-00272-5

**Published:** 2026-08-11

**Authors:** Lijun Jiang, Dan Chen, Qianqian Shi, Tao Huang, Runmao Lin, Jiangzhou Li, Kuai Dai, Chenyang Wang, Wenzheng Ling, Chao Feng, Haichao Cao, Robert Y. L. Wang, Bingyan Xie, Jinguang Yang

**Affiliations:** 1 Key Laboratory of Tobacco Pest Monitoring Controlling & Integrated Management, Tobacco Research Institute, Chinese Academy of Agricultural Sciences, Qingdao, Shandong 266101 China; 2 Shandong Engineering Research Center for Environment-Friendly Agricultural Pest Management, College of Plant Health and Medicine, Qingdao Agricultural University, Qingdao, Shandong 266109 China; 3 Beijing Biomarker Biological Technologies Co., LTD., Beijing, 101300 China; 4 Key Laboratory of Green Prevention and Control of Tropical Plant Diseases and Pests Ministry of Education, College of Plant Protection, Hainan University, Haikou, 570228 China; 5 Yunnan Tobacco Company Yuxi Branch, Yuxi, 653100 China; 6 Department of Biomedical Sciences, Chang Gung University, TaoYuan, 33302 Taiwan; 7 State Key Laboratory of Vegetable Biobreeding, Institute of Vegetables and Flowers, Chinese Academy of Agricultural Sciences, Beijing, 100081 China

**Keywords:** Spatial transcriptomics, Tomato, *Meloidogyne incognita*, Giant cells, Marker genes, Developmental trajectories

## Abstract

Root-knot nematodes (RKNs; *Meloidogyne* spp.) are destructive agricultural parasites, but although giant cell formation is required for establishing parasitism, the mechanism of action has not been fully elucidated. Spatial transcriptomics enables precise spatiotemporal analyses of gene expression, facilitating studies of cell heterogeneity. We performed spatial transcriptomic sequencing on Moneymaker tomato root galls caused by *M. incognita* infection at 3, 5, and 7 days post-inoculation to investigate RKN-induced giant cell formation. Five major cell types were identified; of these, giant cell clusters were localized predominantly in the xylem, stele, and meristem. Four novel giant cell-specific marker genes were confirmed through RNA in situ hybridization. Pseudotime analysis revealed genes potentially associated with giant cell formation. Virus-induced gene silencing (VIGS) of four genes encoding a cyclin-dependent kinase, two cell division cycle-associated proteins, and a MYB3R-1-like transcription factor—hypothesized to maintain the cell cycle or gene expression during mitosis—resulted in significantly fewer galls and significantly smaller giant cells. This study established the first spatiotemporal atlas of RKN-infected tomato roots and identified genes associated with giant cell formation, laying a foundation for further research on the establishment of RKN feeding sites, providing novel insights into RKN pathogenic mechanisms, and potentially guiding novel control strategies.

**Supplementary Information:**

The online version contains supplementary material available at 10.1186/s43897-026-00272-5.

## Core

Five cell types were identified in *M. incognita*-infected tomato roots using spatial transcriptomics; of these, giant cell clusters were localized in the xylem, stele, and meristem. Four novel giant cell-specific marker genes were confirmed. Pseudotime analysis identified three cell cycle-related genes and one MYB3R-1-like transcription factor, whose silencing significantly reduced the number of galls and the sizes of giant cells.

## Gene and accession numbers

Spatial transcriptomic sequencing data can be found in the NCBI Gene Expression Omnibus (GEO) with Bioproject accession number PRJNA1196910 and GEO accession number GSE284052. When the data is in private status, reviewers can access it using the accession number and GEO reviewer token: ulynuaqkljwtrof.

## Introduction

Root-knot nematodes (RKNs; *Meloidogyne* spp.) are destructive parasites that pose a serious threat to agricultural practices (Ibrahim et al. 2011; Jones et al. 2013). During their life cycle, giant cells, metabolically active cells created during nuclear division without cytokinesis, serve as the sole source of nutrients for the developing nematodes (Abad et al. 2009; Bartlem et al. 2014; Grundler and Hofmann 2011). The induction of giant cell formation is important for the establishment of parasitism by RKNs; however, details on the relevant mechanism of action have not been thoroughly elucidated.

When a plant is infected by RKNs, the parasites manipulate key plant biological processes and reprogram plant gene expression (Favery et al. 2016). Studies have implicated CDK (cyclin-dependent kinase) and mitotic cyclin genes in giant cell development (de Almeida et al. 1999); other specific genes include *AtCDKA;1* (Van de Cappelle et al. 2008), *CCS52s* (cell cycle switch gene encoding a 52 kDa protein) and *DEL1* (*DP-E2F-like 1*) (de Almeida et al. 2012; Favery et al. 2002; Koltai et al. 2001), the CDK gene *cdc2a* and the mitotic cyclin gene *cyc1At* (Niebel et al. 1996), and the cyclin-dependent kinase inhibitor gene *KRP6* (*Kip-related protein 6*) (Vieira et al. 2014), among others. Cytoskeleton component (including microtubule and actin filament)-related genes, such as *Arabidopsis thaliana MAP65-3* (*microtubule-associated protein 65–3*) (Caillaud et al. 2008a), the actin genes *ACT2* and *ACT7* (de Almeida et al. 2004), *AtFH6* (*A. thaliana* formin gene *AtFH6*) (Favery et al. 2004), and *ADF2* (*actin-depolymerizing factor 2*) (Clement et al. 2009), are also involved in giant cell ontogenesis. Moreover, multiple hormones (e.g., salicylic acid, jasmonate, ethylene, auxin, cytokinin, and gibberellin) and their biosynthesis-related genes have been reported to play key roles in RKN-induced feeding site formation (Barcala et al. 2010; Bar-Or et al. 2005; Bird and Loveys 1980; Cabrera et al. 2014; De Meutter et al. 2003; Ji et al. 2013; Kyndt et al. 2012).

Despite the identification of thousands of differentially expressed genes in plants linked to successful RKN infection, identifying giant cell-specific genes remains challenging because of cell type heterogeneity within plant tissues (Favery et al. 2016). In multicellular organisms, gene expression is temporally and spatially specific; thus, conventional transcriptomic sequencing is unable to distinguish between individual cells. Single-cell RNA-seq (scRNA-seq) and single-nucleus RNA-seq (snRNA-seq), meanwhile, cannot provide spatial location information, as it is often lost during tissue dissociation, potentially introducing technical artifacts such as stress-induced gene expression. In addition, single-nucleus RNA-seq can capture only the transcripts within the cell nucleus, and thus gene expression information in the cytoplasm may be lost. When plant cells are dissociated into their nuclei, there is a risk of losing certain cell types (such as fragile meristematic cells). Additionally, traditional techniques such as in situ hybridization have difficulty achieving high-throughput detection; they are complex and time-consuming to perform and face additional challenges in the preparation of giant cell protoplasts from RKN infection-induced fibrotic root tissues (Bawa et al. 2022; Giacomello 2021; Hou et al. 2016). Spatial transcriptomics, however, can combine spatial resolution with morphology, thereby allowing the generation of comprehensive gene expression maps and the identification of distinct cell populations while preserving spatial position (Giacomello 2021).

The pioneering application of spatial transcriptomics in plants originated with the generation of high-resolution, spatially resolved transcriptome profiles of model angiosperms and gymnosperms (Giacomello et al. 2017). This technology was subsequently widely applied in multiple fields in botany. For example, the application of SpaTial Enhanced REsolution Omics-sequencing (Stereo-seq) revealed the intricate cell type specificity and characterized the spatial gene expression of the specialized peg organ in peanut (Liu et al. 2022b). Additionally, 10 × Visium technology was used to elucidate the molecular architectural basis of *Phalaenopsis* Big Chili’s floral organogenesis (Liu et al. 2022a). Furthermore, an integrated snRNA-seq and spatial transcriptomics workflow was used to establish a soybean nodule/root cell atlas, revealing that uninfected cells in central infected zones of nodules specialized into functionally distinct subgroups during nodule development and revealing a transitional infected cell subtype enriched with nodulation-related genes (Liu et al. 2023). A spatially resolved transcriptomics approach involving high-throughput analysis of germinating barley grain revealed specific functional gene expression patterns at the subtissue level (Peirats-Llobet et al. 2023). Other recent applications include evaluating the stereoscopic response of rice leaf cells to *Magnaporthe oryzae* infection; flower development and dormancy regulation in peach; and the detailed developmental trajectory of poplar root regeneration (Lv et al. 2024; Wang et al. 2025; Zhao et al. 2025). The successful application of these methods underscores the advantages of spatial transcriptomics in studying the spatial distribution and temporal dynamics of gene expression in various cells, making this technique ideal for deciphering RKN-induced giant cell formation.

In this study, *Meloidogyne incognita*-infected Moneymaker tomato root galls were subjected to spatial transcriptomic sequencing on the third, fifth, and seventh days post-inoculation (dpi). Using previously reported marker genes, five distinct cell types were identified. Of these, giant cell-specific clusters (xylem, stele, and meristem) were distinguished by employing giant cell formation-related genes as marker genes, revealing differentially expressed genes (DEGs) between giant cells and normal cells. Four DEGs were newly confirmed to be giant cell-specific markers via spatial expression analysis and RNA in situ hybridization. Differentiation analysis of the cell-type profiles of giant cell-specific clusters was then performed through pseudotime trajectory analysis, identifying key genes potentially associated with the formation of giant cells. The silencing of three upregulated genes related to the cell cycle and division and of one transcription factor that potentially regulates gene expression during mitosis resulted in reduced numbers of galls and smaller giant cells after RKN infection. Our research identified specific giant cell types through the analysis of spatial transcription profiles in *M. incognita*-infected tomato roots and validated novel giant cell markers and critical genes related to giant cell formation, thus providing a significant theoretical advance in elucidating RKN parasitic mechanisms.

## Results

### Spatial transcriptomic sequencing of RKN-infected tomato roots

The root galls of *M. incognita*-infected Moneymaker tomato plants were subjected to spatial transcriptomic profiling at 3, 5, and 7 days post-inoculation with the BMKMANU S1000 platform. After quality filtering, we obtained clean reads of 336,789,621 (101.04 G), 501,573,660 (150.47 G), and 563,761,625 (169.13 G) for the three samples, respectively (Table S1). The tissue sections were stained with toluidine blue (Fig. S1), after which gene expression was quantified via unique molecular identifiers (UMIs), and valid barcodes and UMIs were analyzed using BSTMatrix (Table S1). The read mapping rates to the Moneymaker tomato reference genome were 97.07%, 94.99%, and 95.26%, while 86.02%, 82.30%, and 82.12%, respectively, of the reads were supported by the GTF information of the transcripts (Table S1).

### Identification of spot clusters

We then analyzed spatial gene expression matrices with adjusted supspot parameters (Table S2). The spatial distributions and UMI/gene profiles of the spots are shown in Fig. S2. At level 6, the three samples contained 1,835, 3,883, and 8,067 supspots, along with corresponding median UMIs of 4,865, 1,060, and 564, median genes per supspot of 2,561, 760, and 441, and total detected genes of 22,512, 22,858, and 23,379, respectively (Tables S2–S3).

**Fig. 1:**
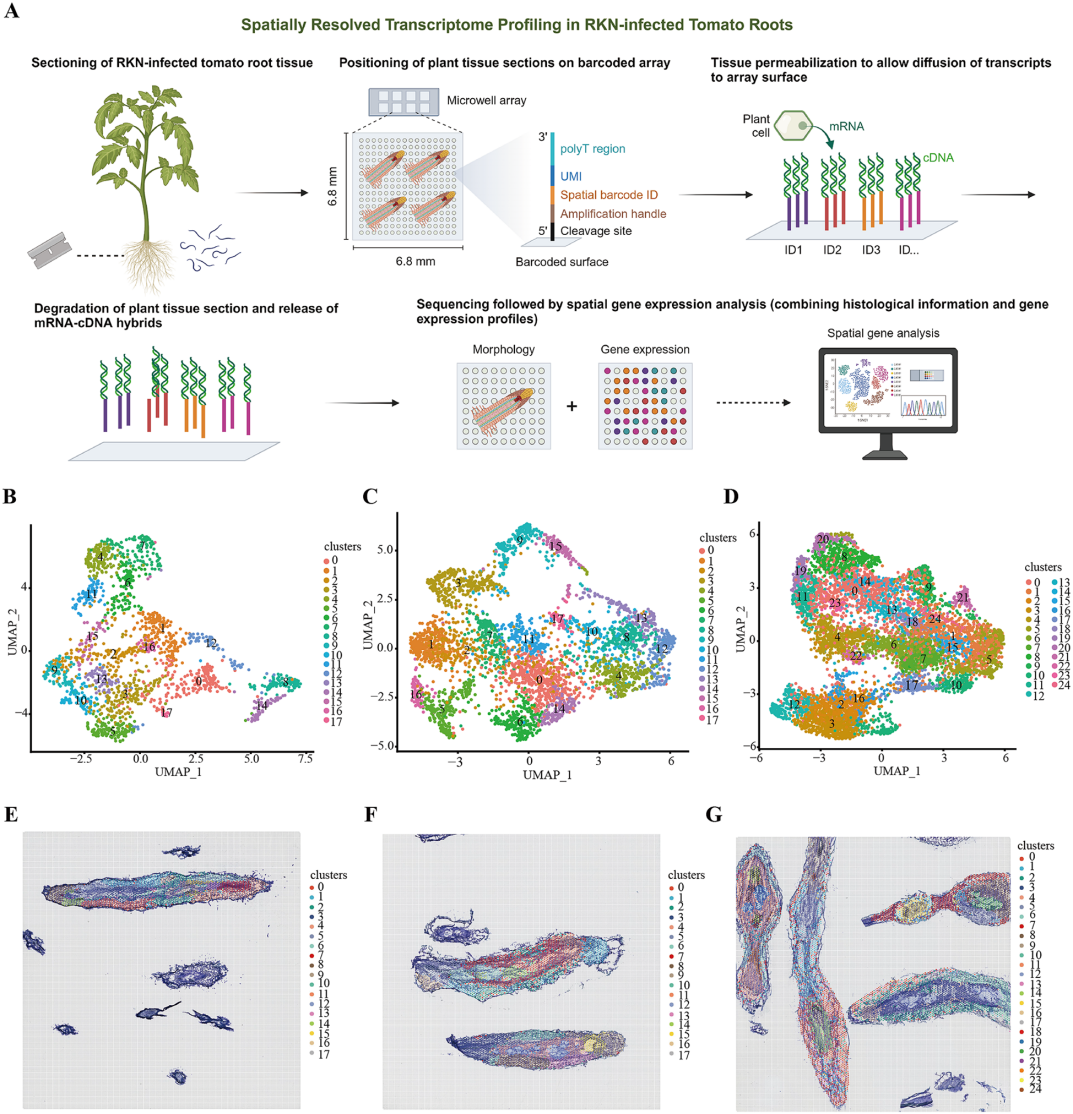
Workflow for the spatial transcriptomic sequencing of *M. incognita*-inoculated Moneymaker tomato roots on a BMKMANU S1000 alongside visualization of spot clusters and cluster distribution according to uniform manifold approximation and projection (UMAP). **A** Workflow for the sequencing of *M. incognita*-inoculated Moneymaker tomato roots on a BMKMANU S1000. **B**-**D**. UMAP of spot clusters of tissue sections on the third, fifth, and seventh days post-inoculation. Each point corresponds to a spot, with different colors denoting distinct clusters. **E**–**G** Distribution plots of spatial region clusters across distinct supspots at multi-resolution levels of tissue sections collected at 3, 5, and 7 days post-inoculation (dpi). Each point corresponds to a spot, and different colors denote distinct clusters

Uniform manifold approximation and projection (UMAP) dimensionality reduction identified 18, 18, and 25 spatial clusters in the samples collected at days 3, 5, and 7 post-inoculation, respectively (Fig. 1). The cluster distribution is illustrated in Fig. 1 and Figs. S3–S5, and the average gene expression across all the clusters is summarized in Table S4. The top 10 feature genes of each cluster are listed in Tables S5–S7 and are visualized in Figs. S6–S9.

### Cluster annotation according to reported marker genes

RKNs induce giant cell formation across diverse host plants via a conserved mechanism. Accordingly, we annotated cell clusters using homologous cell-type marker genes from multiple species. We first retrieved candidate markers from two well-established databases, PlantscRNAdb (http://ibi.zju.edu.cn/plantscrnadb/) and PlantCellMarker (https://www.tobaccodb.org/pcmdb/homePage), to guarantee the identification of reliable and comprehensive gene sets. Next, we conducted BLASTP homology searches between the Moneymaker genome and the reference genomes of *A. thaliana* (TAIR10, https://www.arabidopsis.org/), *Solanum lycopersicum* (SL3.0.58, https://plants.ensembl.org/index.html), *Oryza sativa* (MSU_v7, https://rice.uga.edu/download_osa1r7.shtml; IRGSP_1.0, https://plants.ensembl.org/index.html), *Nicotiana tabacum* (Nitab_v4.5, http://solgenomics.net/ftp/genomes/Nicotiana_tabacum/edwards_et_al_2017) and *Zea mays* (B73_RefGen_v4, https://plants.ensembl.org/index.html) under the screening condition ‘-e 1e-5’. Reciprocal best hits (RBHs) were defined as optimal homologs, and the best hits from BLAST were defined as suboptimal matches (labeled Blast), verifying the functional conservation of the screened markers in Moneymaker tomato. Based on the homology and expression results, we selected specific marker sets for each sample. For the 3 dpi sample, 31 database-derived markers yielded 28 Moneymaker homologs, whereas 28 markers (yielding 25 Moneymaker homologs) and 30 markers (yielding 25 Moneymaker homologs) were applied to the 5 dpi and 7 dpi samples, respectively (Table S8).

**Fig. 2:**
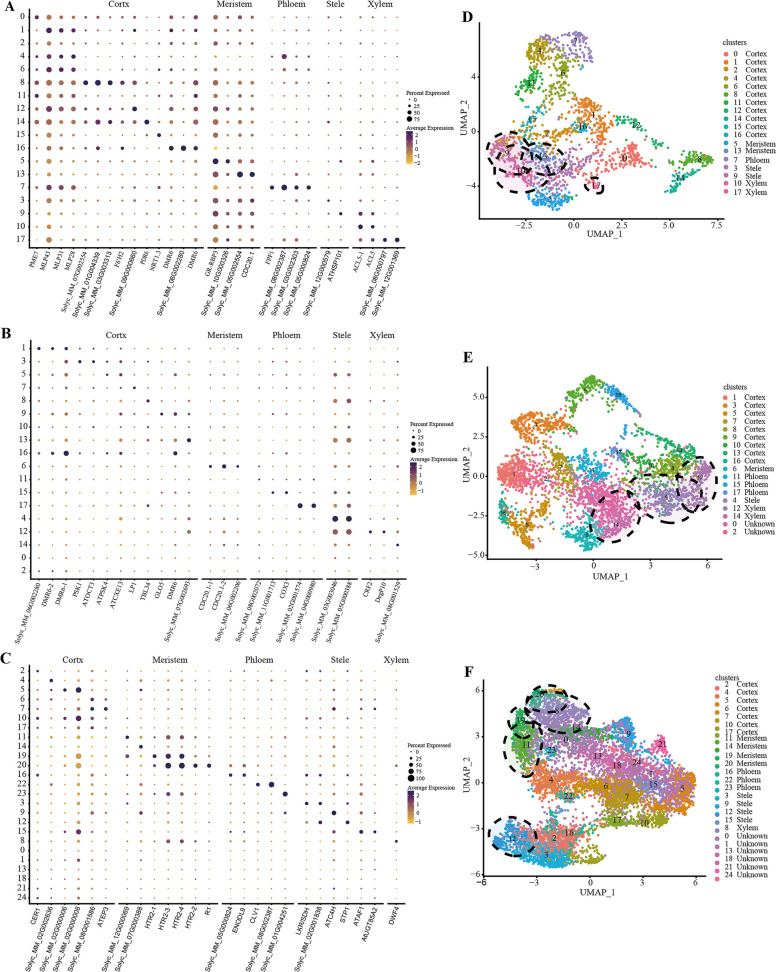
Dot plots of marker genes and UMAP of the three tissue samples generated for cell type identification. **A**-**C** Dot plots of marker genes for cell type identification in samples on the third, fifth, and seventh days are shown in panels A, B, and C, respectively. The z-score of the average expression level of the marker genes is denoted by the dot color, with the cell percentage indicated by the dot size. **D**-**F** UMAP of spatial spots for clusters identified in tissue samples harvested at 3, 5, and 7 days post-inoculation (dpi). The points correspond to individual spots, and the clusters are labeled with different cell types and colors. The dotted lines define clusters to which the giant cells belong

Combining histological examination and spatial gene expression distribution information (Fig. 2, Table S8), we annotated clusters according to the following core logic: a cluster was assigned to a specific cell type if the corresponding homologous markers in Moneymaker were highly and specifically expressed. For the 3 dpi sample, the homologous tomato genes of reported meristem markers, *Solyc_MM_10G000326* and *Solyc_MM_10G002333* (*GR-RBP3*), were uniquely expressed in cluster 5. In contrast, *Solyc_MM_05G002554* and *Solyc_MM_06G002207* (*CDC20.1*) were specifically expressed in cluster 13. These two clusters were thus inferred to represent meristem cells. The expression patterns of the homologous phloem markers, *Solyc_MM_01G002015* (*FPF1*), *Solyc_MM_03G002303*, *Solyc_MM_05G000824*, and *Solyc_MM_08G002387* in cluster 7 indicated the presence of phloem cells. Clusters 3 and 9 were identified as stele cells, as evidenced by the obvious expression of *Solyc_MM_12G000579* and *Solyc_MM_03G002652* (*ATHSP101*). Cluster 10 was identified as representing xylem cells because of the preferential expression of *Solyc_MM_08G001154* (*ACL5*) and *Solyc_MM_09G002242* (*ACL5*); cluster 17 was classified as the same cell type based on the elevated expression of *Solyc_MM_08G000797* and *Solyc_MM_12G001369*. Finally, clusters 0, 1, 2, 4, 6, 8, 11, 12, 14, 15, and 16 were designated as cortex cells using the same methodology, while the same criteria were applied when we correspondingly annotated cell types for the 5 dpi and 7 dpi samples.

**Fig. 3:**
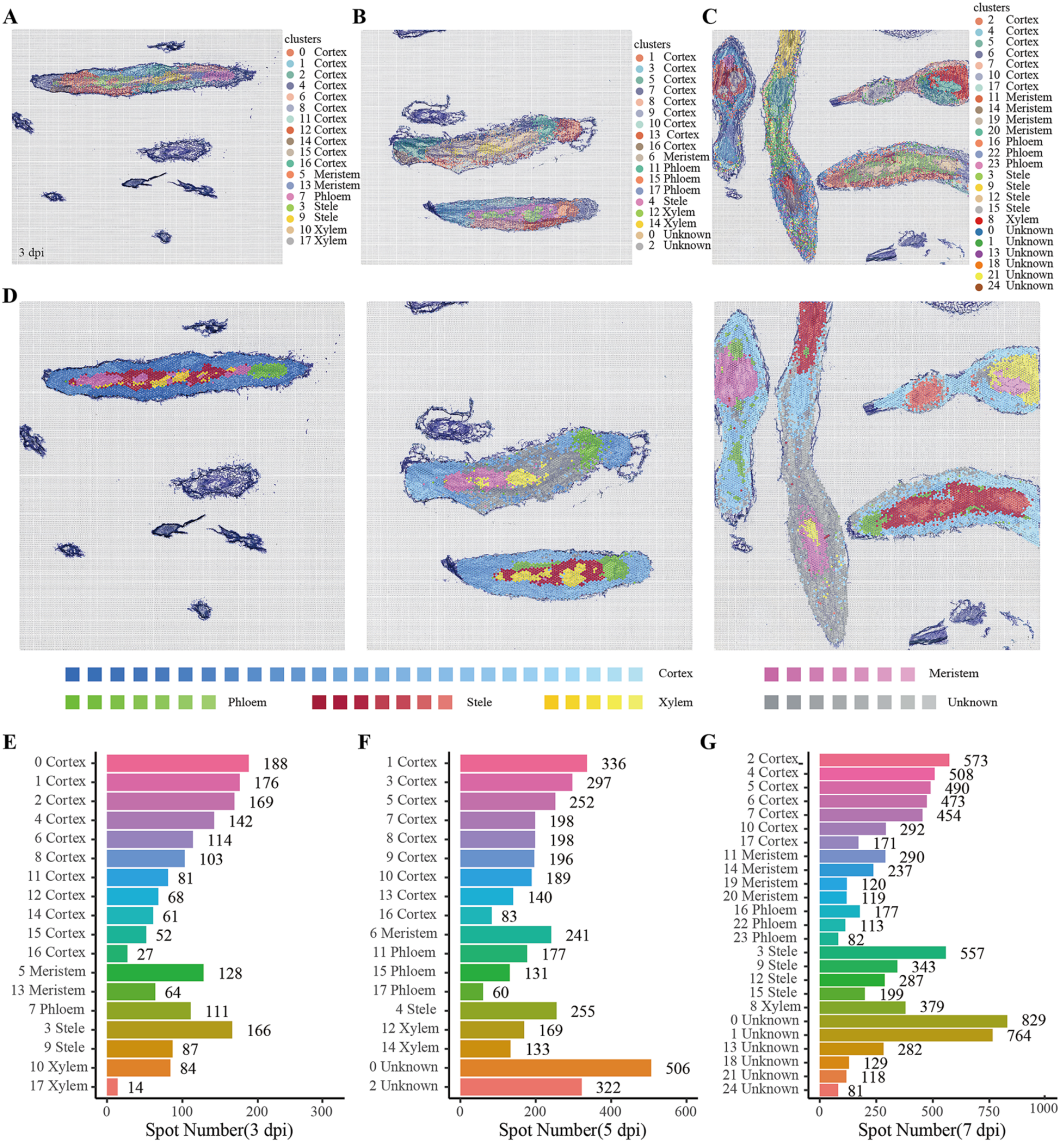
Cell types discovered in spatial slice samples from the three different time points, and statistical data of the spot numbers. **A** Cell type annotation for the spatial slice sample on Day 3 post-inoculation. **B** Cell type annotation for the spatial slice sample on Day 5 post-inoculation. **C** Cell type annotation for the spatial slice sample on Day 7 post-inoculation. **D** Cell type annotation for the combination of samples from three time points (3, 5, and 7 days post-inoculation). The points correspond to the spatial spots, and different colors indicate different cell types. **E**–**G** Histogram of spot numbers in each cluster for samples at the three time points in (**D**)

In total, across the three time points, we identified five major cell types in *M. incognita*-infected tomato root galls: cortex (27 clusters), meristem (7 clusters), phloem (7 clusters), stele (7 clusters), and xylem (5 clusters). Eight clusters were classified as “unknown” because the marker genes used to identify the cell types could not be used to uniquely define them (Fig. 3A–D). Further analysis revealed that these clusters had extremely low numbers of detected genes and poor data quality (Fig. S10). Combined with tissue morphology, we inferred that these clusters corresponded to hollow regions caused by nematode infection and were therefore excluded from subsequent analysis. The spot counts of all the clusters from the three samples are summarized in Fig. 3E–G.

### Identification of giant cell-specific clusters

To identify clusters corresponding specifically to giant cells, we selected *A. thaliana* marker genes proven to regulate the formation and development of RKN feeding sites. These genes include *ARF5* (*AUXIN-RESPONSIVE-FACTOR-5*), *AtIAA12*, *AtIAA28*, *SHR* (*SHORT-ROOT*; root apical meristem regulator), *AtCCS52* (associated with feeding site formation) (de Almeida et al. 2012; Olmo et al. 2020), *Atcdc2a*, *Atcyc1* (mitotic cyclin; related to early feeding cell formation), *MAP65-3* (related to giant cell ontogenesis) (Caillaud et al. 2008b; Niebel et al. 1996), *ADF2* (*actin-depolymerizing factor 2*; direct giant cell formation) and *CKS2* (overexpressed in 7-day galls) (Clement et al. 2009; Jammes et al. 2005). Their tomato homologs were identified via RBH and BLAST (Table S9) and used for cluster annotation.

**Fig. 4:**
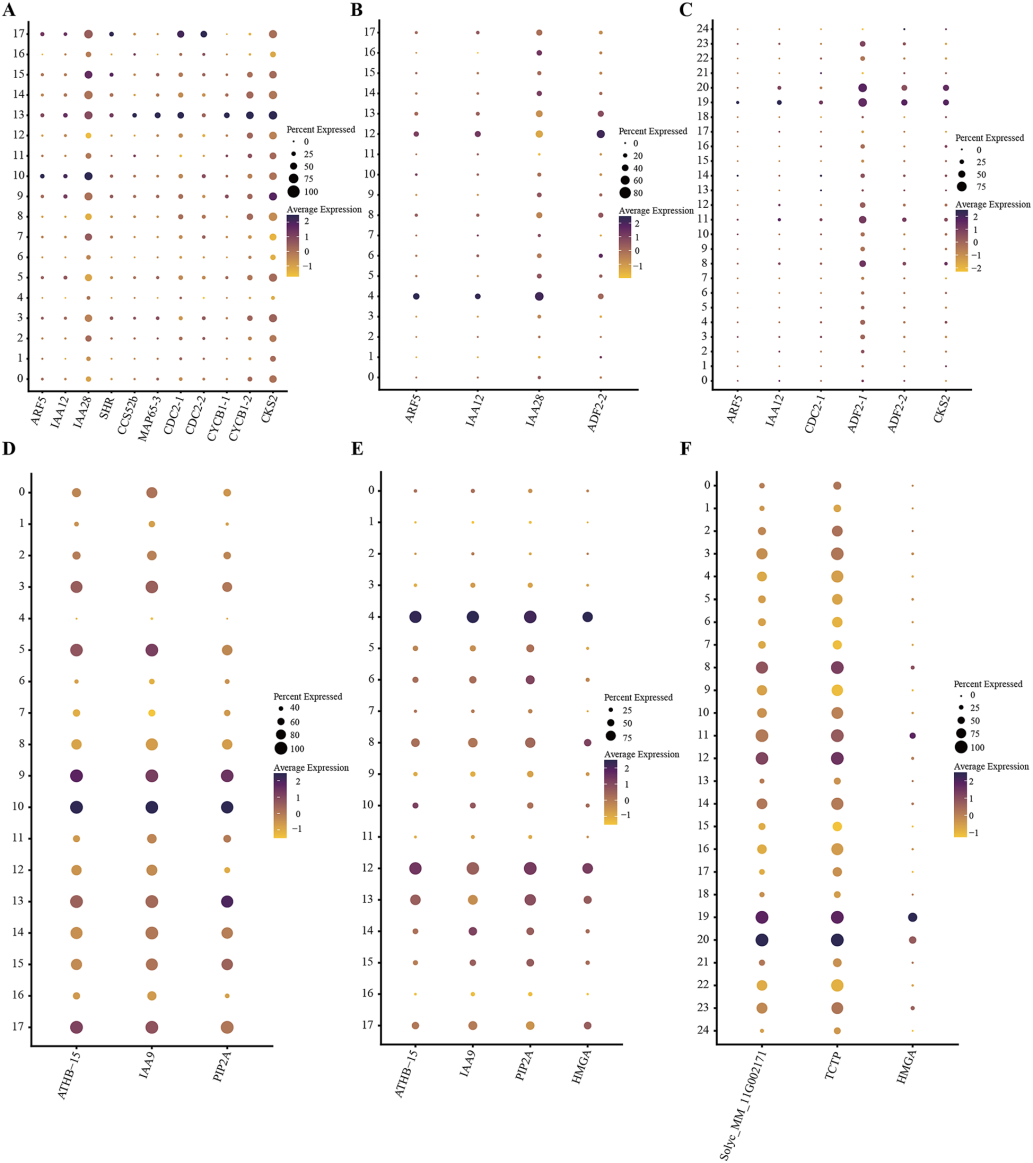
Dot plots of candidate marker genes for giant cell identification and the newly discovered potential marker genes. **A**-**C** Dot plots of candidate marker genes for identifying giant cell-specific clusters from samples harvested at 3 (**A**), 5 (**B**), and 7 (**C**) days post-inoculation (dpi). **D**-**F** Dot plots of potential novel marker genes identified from giant cell-specific clusters in samples harvested at 3 (**D**), 5 (**E**), and 7 (**F**) days post-inoculation (dpi). The color of each dot indicates the z-score of the average expression level of the corresponding marker genes, while the size of each dot reflects the percentage of cells expressing that marker gene

We profiled the expression of these homologs across the three time points. For the Day 3 sample, the auxin-responsive factor homolog *Solyc_MM_04G002785* (*ARF5*) exhibited elevated expression levels in clusters 10, 13, and 17; *Solyc_MM_09G002537* (*IAA12*) displayed high expression in clusters 9, 10, 13, and 17; *Solyc_MM_03G003224* (*IAA28*) was abundantly expressed in cluster 10. For the Day 5 sample, *Solyc_MM_04G002785* and *Solyc_MM_09G002537* were highly expressed in clusters 4 and 12, and *Solyc_MM_03G003224* was highly expressed in clusters 4 and 14. For the Day 7 sample, *Solyc_MM_04G002785* was highly expressed in cluster 19, and *Solyc_MM_09G002537* was highly expressed in clusters 8, 11, 12, 19, and 20. Additionally, *Solyc_MM_04G000471* (*ADF2-2*) demonstrated elevated expression in cluster 12 in the Day 5 sample and in clusters 8, 11, 19, and 20 in the Day 7 sample, while *Solyc_MM_06G000035* (*ADF2-1*) displayed elevated expression in clusters 8, 11, 12, 19, and 20 in the Day 7 sample. Furthermore, *Solyc_MM_08G001365* (*CDC2-1*) was highly expressed in clusters 13 and 17 in the Day 3 sample and in clusters 11 and 19 in the Day 7 sample, while *Solyc_MM_12G002371* (*CDC2-2*) was highly expressed in cluster 17 in the Day 3 sample. *Solyc_MM_11G002008* (*CKS2*) exhibited high expression in clusters 9 and 13 in the Day 3 sample, as well as in clusters 8, 11, 19, and 20 in the Day 7 sample. In addition, in the Day 3 sample, *Solyc_MM_01G000359* (*CYCB1-1*), *Solyc_MM_10G002312* (*CYCB1-2*), *Solyc_MM_06G000850* (*CCS52b*) and *Solyc_MM_03G000205* (*MAP65-3*) showed high expression in cluster 13, while *Solyc_MM_02G003216* (*SHR*) was found to be highly expressed in cluster 17. The consistently high expression of giant cell signature genes in the above clusters confirms their identity as giant cell-specific clusters. The overall expression patterns of these homologs across all clusters are presented in Fig. 4A–C, with their spatial distribution shown in Fig. S11.

In conclusion, we successfully identified giant cell-specific clusters across three time points: the 3 dpi sample included clusters 9 (stele), 10 (xylem), 13 (meristem), and 17 (xylem); the 5 dpi sample contained clusters 4 (stele), 12 (xylem), and 14 (xylem); and the 7 dpi sample harbored clusters 8 (xylem), 12 (stele), and 11, 19, 20 (meristem) (Fig. 4A–C). These clusters are highlighted with dotted lines in the UMAP plots (Fig. 2). Unlike a recent snRNA-seq study in which giant cell types were reportedly derived from xylem and stele cells (Zadegan et al. 2025), our work further revealed meristem-derived giant cell clusters, which support the view that meristematic cells exhibit transient pluripotency during gall organogenesis (Olmo et al. 2020). To validate these annotations, GO enrichment analysis of upregulated DEGs (fold change ≥ 1.5, p-value < 0.05) between giant cell clusters and the other clusters was performed (Table S10); representative and significant GO terms (p-value < 0.05) are shown in Fig. S12. In the Day 3 sample, genes were enriched in plant hormones (auxin, cytokinin, and gibberellin) (GO:0009739, GO:0009735, GO:0009733, and GO:0009725), root development regulation (GO:2,000,280), and nutrient transport and metabolic processes (GO:0006006 and GO:0006869, among others). Cell wall organization (GO:0009664) and biogenesis (GO:0042546) were predominant in the enriched genes identified in the 5 and 7 dpi samples, whereas genes enriched in DNA replication (GO:0006260) were revealed in cluster 20 in the 7 dpi sample. Oxidative stress response (GO:0034599) and transport-related terms were universally enriched across all groups. The identification of these active synthesis and metabolism features verify the identity of the annotated giant cell clusters.

In this study, cross-species homologous markers originally characterized in healthy roots may lose expression specificity due to nematode-induced cellular reprogramming and tissue distortion in galls, thereby reducing the reliability and independence of the cell annotation. To address this potential limitation, we conducted single-gene spatial visualization and gene set scoring via the “AddModuleScore” function in the Seurat R package with default parameters. The results revealed that individual and groups of tissue markers maintained region-restricted expression in both normal tissues and infected lesions (Figs. S13-S18). These findings confirm that the selected markers exhibit conserved spatial expression patterns and validate the reliability of our cluster and cell type annotations.

**Fig. 5:**
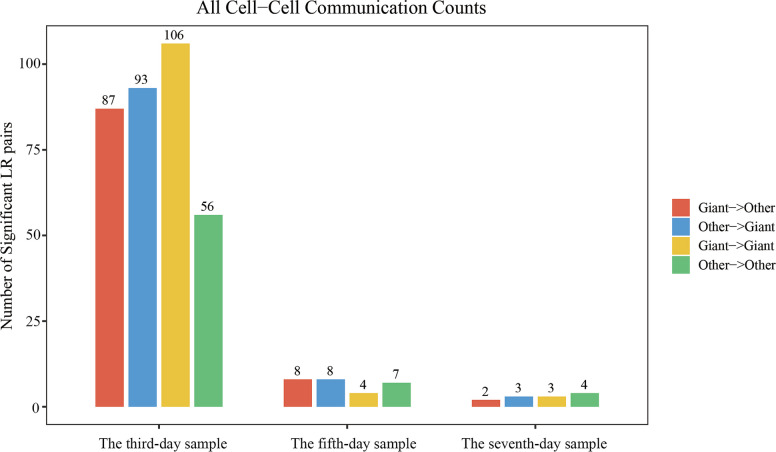
Histogram of cell–cell communication counts between giant cells and their surrounding cells. The horizontal coordinate represents the sample names, while the vertical coordinate indicates the number of significant LR (ligand-receptor) pairs

We explored the cell–cell communication between giant cells and adjacent cells to investigate the signal transmission and exchange between receptors and ligands (Table S11). The total number of significant ligand-receptor (LR) pairs exhibited an extremely significant and cliff-like decline from 3 dpi to 5 dpi and 7 dpi (Fig. 5). This result indicates that at the early stage of infection (3 dpi), newly formed giant cells underwent active reprogramming, cell wall remodeling, nutrient transport, and signal recruitment. The abundant LR interactions facilitated intense signal and material exchange, providing a molecular basis for giant cell differentiation and expansion and parasitic microenvironments for the establishment of feeding sites. At the middle stage of nematode infection (5 dpi), the morphology and expansion of the giant cell had largely stabilized, accompanied by reduced cellular communication. By 7 dpi (late infection stage), the giant cells had achieved full functional specialization and finalization of their characteristics, becoming dedicated nutrient reservoirs for nematodes, with LR pair counts decreasing to their lowest levels. This trend reflects the dynamic changes in intercellular cross-talk throughout infection progression.

### Discovery of novel giant cell marker genes and verification with RNA in situ hybridization

The DEGs between giant cell-specific clusters and the other clusters were analyzed (fold change ≥ 1.5, p-value < 0.05); detailed gene expression and annotation information is provided in Table S12. For the Day 3 and 5 samples, four genes were selected for expression pattern analysis (Table S12): *Solyc_MM_12G001860* (*ATHB-15*), a homeobox-leucine zipper protein-encoding gene involved in procambium and xylem cell differentiation (Ohashi-Ito and Fukuda 2003); *Solyc_MM_04G002372* (*IAA9*), an AUX/IAA protein-encoding gene essential for gall formation (Olmo et al. 2020); *Solyc_MM_09G000177* (*PIP2A*), an *aquaporin* gene that regulates water transport and root growth (Ding et al. 2019); and *Solyc_MM_05G002120* (*HMGA*), a gene that encodes an HMG-Y-related B protein that functions as an architectural elements for modifying DNA and chromatin structure (Bustin 1999). For the Day 7 sample, three genes were chosen (Table S12): *Solyc_MM_11G002171*, which encodes a eukaryotic peptide chain release factor GTP-binding subunit-like protein that may be involved in mitosis (Na et al. 2014); *Solyc_MM_01G003484* (TCTP), which encodes a translationally controlled tumor protein homolog and is potentially linked to cell proliferation (Tao et al. 2015); and *Solyc_MM_05G002120* (HMGA), which encodes an HMG-Y-related B protein that mediates DNA and chromatin remodeling.

**Fig. 6:**
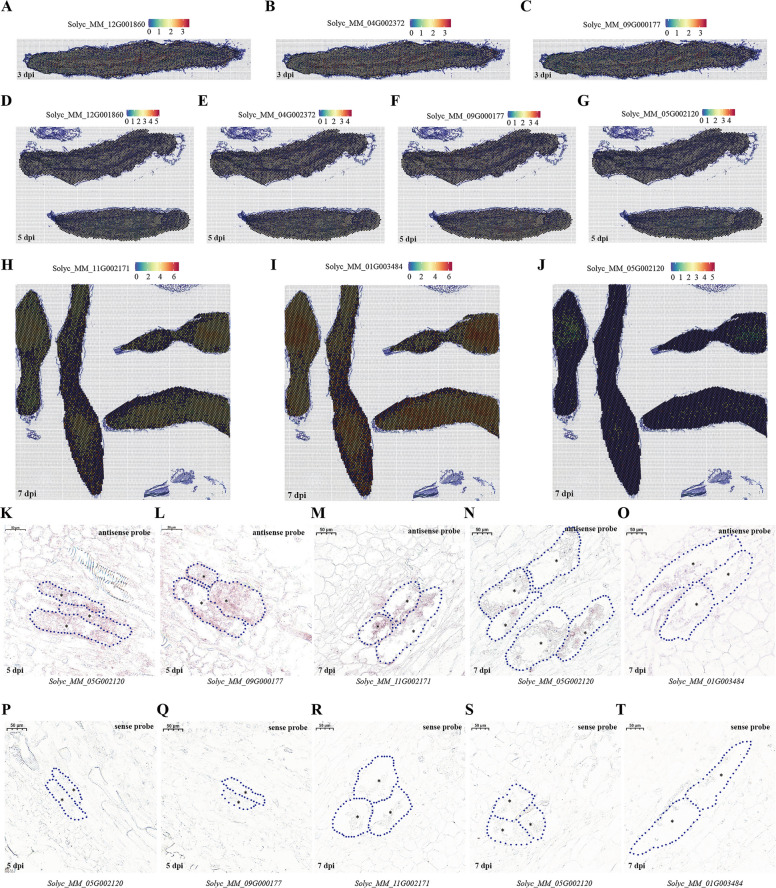
Spatial expression distribution of potential novel marker genes for giant cell and RNA in situ hybridization verification. **A**-**J** Spatial expression distribution maps of potential giant cell marker genes for the samples from three time points (3, 5, and 7 days post-inoculation). Each point represents a spatial spot. The colors denote the gene expression levels; red indicates a high expression level, while blue indicates a low expression level. **K**-**L** RNA in situ hybridization (ISH) validated the presence of transcripts from *Solyc_MM_05G002120* and *Solyc_MM_09G000177* within giant cells in tissue sections of galls from *M. incognita*-infected tomato roots harvested at 5 days post-inoculation (5 dpi). **M**-**O** RNA in situ hybridization (ISH) validated the presence of transcripts from *Solyc_MM_11G002171*, *Solyc_MM_05G002120*, and *Solyc_MM_01G003484* within giant cells in tissue sections of galls from *M. incognita*-infected tomato roots harvested at 7 days post-inoculation (7 dpi). The asterisks represent the giant cells. **P**-**T** Analysis of negative controls using sense RNA probes for potential novel marker genes, demonstrating hybridization specificity. The asterisks indicate the giant cells, and the outlines of the giant cells are circled

Dot plot analysis revealed distinct expression patterns of the candidate markers across samples (Fig. 4D–F). At 3 dpi, *Solyc_MM_12G001860* and *Solyc_MM_04G002372* were enriched in clusters 9 and 10, whereas *Solyc_MM_09G000177* was highly expressed in clusters 9, 10, and 13 (Fig. 4D). All four selected genes showed prominent expression in clusters 4 and 12 at 5 dpi (Fig. 4E). At 7 dpi, *Solyc_MM_11G002171* were predominantly detected in clusters 8, 12, 19, and 20; the same was true for *Solyc_MM_01G003484*, which was also predominantly expressed in cluster 11. *Solyc_MM_05G002120* was highly expressed in clusters 11, 19, and 20 (Fig. 4F). All these clusters were previously defined as giant cell clusters, and these spatial expression profiles further supported their consistency with the localization of the giant cell cluster (Fig. 6A–J and Fig. S19).

RNA in situ hybridization was performed on *M. incognita*-infected tomato gall sections to confirm the expression of the candidate giant-cell marker genes (Fig. 6K–T). Given the overlapping gene expression in the Days 3 and 5 samples and stable developmental phases of giant cells facilitating signal detection, 4 genes (*Solyc_MM_12G001860*, *Solyc_MM_04G002372*, *Solyc_MM_09G000177*, and *Solyc_MM_05G002120*) identified in the Day 5 sample and 3 genes (*Solyc_MM_05G002120*, *Solyc_MM_11G002171*, and *Solyc_MM_01G003484*) in the Day 7 sample were selected for further analysis. Consequently, *Solyc_MM_09G000177* and *Solyc_MM_05G002120* were found to be strongly and specifically expressed in the giant cells in the 5 dpi sections (Fig. 6K–L, P–Q). Similarly, *Solyc_MM_05G002120*, *Solyc_MM_11G002171*, and *Solyc_MM_01G003484* displayed distinct giant cell signals in the 7 dpi sections (Fig. 6M–O, R–T). These results confirm that these genes are credible giant cell markers and verify our spatial transcriptome data.

We next compared our giant cell-specific (upregulated) genes with 772 candidates from a published snRNA-seq study (Zadegan et al. 2025) (Table S12). Venn diagram analysis identified 30, 20, and 11 overlapping DEGs in our 3, 5, and 7 dpi samples, respectively (Fig. S20A–C). We performed the overlapping gene set scoring analysis using the “AddModuleScore” function in the Seurat R package with default parameters. These shared genes exhibited collaborative high expression in giant cell regions (Fig. S20D–F), indicating their potential as universal giant cell markers. Given the inconsistent sampling time points between the two studies, this hypothesis requires further validation in future studies.

### Differentiation analysis of cell-type profiles in giant cell-specific clusters via pseudotime trajectory

Giant cells are induced and differentiated from vascular parenchyma cells and meristem cells with differentiation potential within vascular bundle tissue after RKN second-stage juveniles invade the host roots. In this study, pseudotime trajectory analysis using Monocle 2 was employed to explore the differentiation trajectories and states of four vascular bundle cell types (meristem, stele, phloem, and xylem) in three tissue samples to identify key genes related to giant cell formation.

**Fig. 7:**
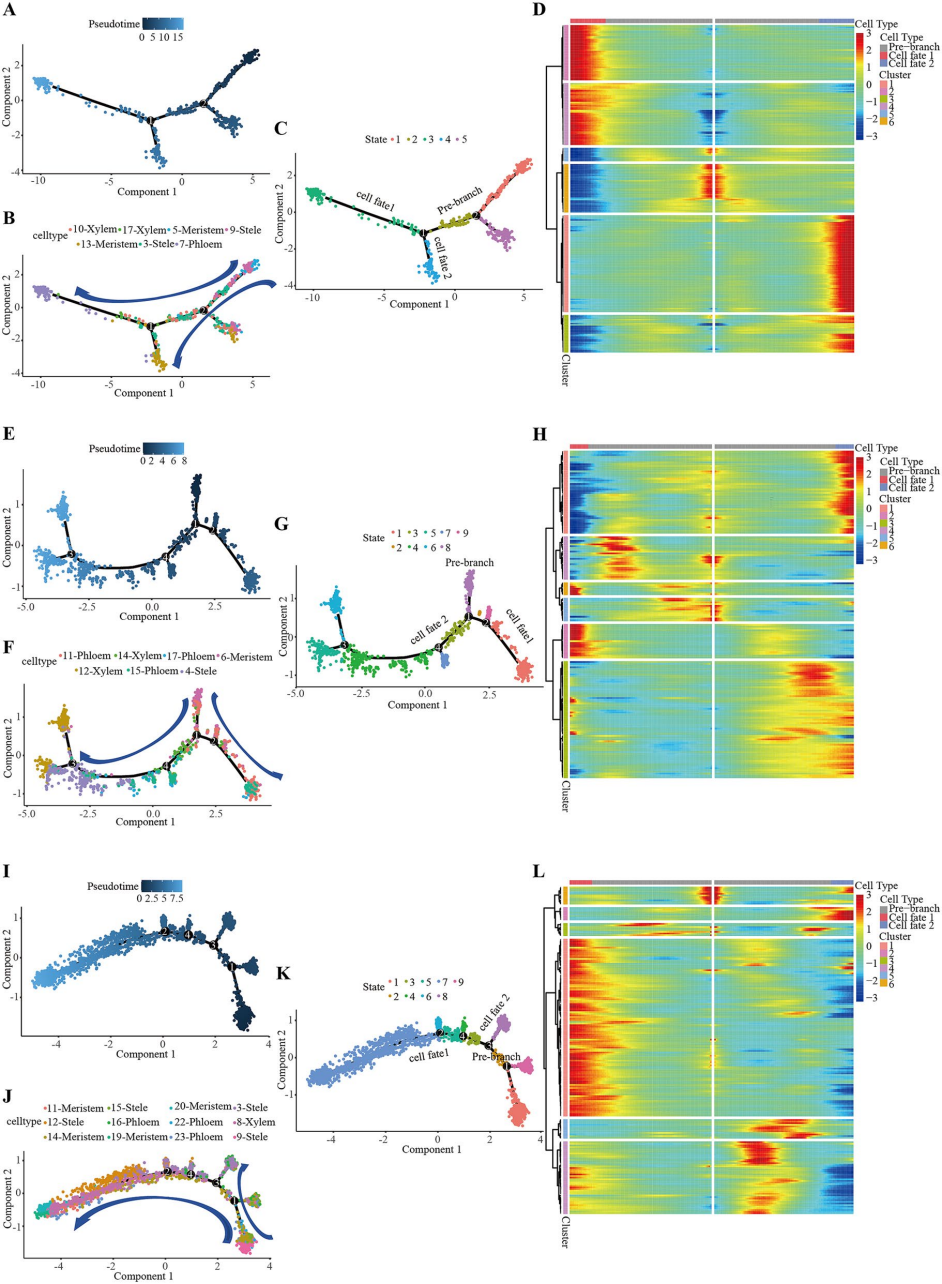
Pseudotime trajectory of the differentiation of vascular bundle cells into giant cells. **A**, **E**, **I** Pseudotime distribution of vascular bundle cells in tissue slice samples collected at 3, 5, and 7 days post-inoculation (dpi). The horizontal and vertical coordinates represent the components after dimensionality reduction using DDRTree. Different branches represent different evolutionary or differentiation trajectories. Each dot represents a spatial spot, colored according to pseudotime values as shown by the scale bar. Darker blue indicates a smaller pseudotime value, corresponding to an earlier development/differentiation state. **B**, **F**, **J** The same trajectory colored by cluster/cell type, showing the distribution of vascular cell populations along the differentiation paths. Different colors denote different clusters. **C**, **G**, **K** The same trajectory colored by cell state. Different colors represent different cell states. **D**, **H**, **L** Heatmap of BEAM analysis results showing the dynamics of differential gene expression along pseudotime between the differentiation states of giant cells and normal vascular bundle cells. Genes are grouped into distinct clusters on the basis of their pseudotime-dependent expression patterns

According to the cell differentiation trajectories, for the Day 3 sample, the vascular bundle cells differentiated into two directions at key node 1: cell fate 1 (normal vascular bundle cells; cluster 7, Phloem) and cell fate 2 (giant cells; cluster 13, Meristem) (Fig. 7A–C). A similar bifurcation was observed in the Day 5 sample at key node 1: cell fate 1 (normal vascular bundle cells; clusters 11 and 15, Phloem) and cell fate 2 (giant cells; cluster 4, Stele, and cluster 12, Xylem) (Fig. 7E–G). For the Day 7 sample, the differentiation trajectory of vascular bundle cells bifurcated at key node 3: cell fate 2 (normal vascular bundle cells; clusters 9 and 15, Stele, and cluster 22, Phloem) and cell fate 1 (giant cells; cluster 8, Xylem; clusters 11, 19, and 20, Meristem; and cluster 12, Stele) (Fig. 7I–K).

BEAM heatmaps showed the DEGs (qval < 1e-4) between the two fates were classified into six clusters for all the samples. For the Day 3 sample (node 1), cluster 1 (72 genes) and cluster 3 (28 genes) were closely associated with the differentiation of cell fate 2 (Fig. 7D and Table S13). For the Day 5 sample (node 1), cluster 1 (46 genes) was associated with the differentiation of cell fate 2 (Fig. 7H and Table S13). For the Day 7 sample (node 3), cluster 1 (110 genes) was linked to the differentiation of cell fate 1 (Fig. 7L and Table S13).

To identify key genes that regulate giant cell formation, we analyzed the DEGs that potentially participate in vascular bundle cell differentiation at the branch points (Table S13). Normally, the plant mitotic cell cycle progresses through the G1, S (synthesis), G2, and M (mitosis) phases. Giant cells originate from vascular binucleate cells via cell cycle activation, followed by repeated, truncated cycles to generate large multinucleated cells. Analysis of the Day 3 sample revealed that the DEGs included those encoding cyclin-dependent kinases (critical for cell cycle regulation) (Andersen et al. 2008; Van de Cappelle et al. 2008) and G2/mitotic-specific cyclins (early-induced in galls and the feeding sites during the G2 phase) (de Almeida et al. 1999). CCS52B, which encodes a division cycle-associated protein (Goh et al. 2000; Niu et al. 2015), and mitosis regulator MYB3R-1-like (Kobayashi et al. 2015), also came to light. These genes may act at the initial stage of giant cell development. RKN-induced giant cells act as permanent nutrient reservoirs, and their development relies on acytokinetic nuclear division, continuous cell expansion, nutrient transport, and cytoskeleton remodeling. At the early stage, the early nodulin-like protein, which is potentially involved in nutrient transport and growth modulation (Ntui et al. 2024), the tubulin alpha chain-like protein, which typically accompanies plant cell division and elongation (Pastuglia and Bouchez 2007), and the aquaporin were detected. It is presumed that the genes encoding these proteins may function as potential regulators of giant cell formation.

Multinucleated giant cell formation is inseparable from the expression of related genes in plants. The genes identified in the Day 5 sample included those that encode the transcription factor for cell proliferation and development (Sozzani et al. 2010; Zhao et al. 2024); the eukaryotic translation initiation factor, which modulates cell division, growth, and death and is critical for plant growth and development (Feng et al. 2007; Hossain et al. 2012; Thompson et al. 2004); and the translation elongation factor potentially involved in plant development (Hossain et al. 2012) (Table S13). Cell growth and DNA replication occur concurrently during giant cell development. In the Day 7 sample, we identified high mobility group (HMG) protein genes regulating DNA replication and gene expression (Singh and Rajeswari 2001), the AUX/IAA genes governing plant growth and development (Salehin et al. 2015), and genes encoding translationally-controlled tumor protein homologs facilitating in vitro plant regeneration (Toscano-Morales et al. 2015) (Table S13). The detection of the tubulin beta chain gene further proves that cytoskeleton regulation occurs throughout this process (Table S13). The nucleosome is the basic unit of chromatin and serves as a fundamental regulatory unit in DNA-templated processes; as the host cells transform into giant cells, nucleic acid and protein levels increase exponentially. Histone-related protein genes, which form the nucleosome (Foroozani et al. 2022), were identified in the Day 7 sample (Table S13). The identification of these genes genetically reveals that the later-stage giant cell formation is accompanied by interference with DNA replication, gene expression, cell division, and development processes.

To more intuitively illustrate the role that these genes potentially play in giant cell formation, the dynamics of their expression were further analyzed and are presented in Fig. S21. The expression patterns of these genes in giant cells and normal vascular bundle cells exhibited opposite trends, verifying their certain regulatory functions in giant cell development and formation. We further compared our results with the data from a recent single-nucleus RNA-seq study. Many cell cycle regulation and tubulin alpha chain-like genes detected in the Day 3 sample overlapped with those reported in prior datasets (Table S14), which is consistent with and supports the previous conclusion that young giant cells specifically expressed genes involved in the mitotic cell cycle and related processes and that the microtubule-binding protein TPX2 and B-type cyclins are highly expressed in early pseudotime (Zadegan et al. 2025). Such overlap confirms the reliability of our finding to some extent. Moreover, numerous genes from our Day 5 and Day 7 samples did not overlap with those in prior research (Table S14). All candidate genes identified in this work require further experimental verification.

### Functional validation of giant cell formation initiation-related genes via VIGS

To explore the genes associated with giant cell formation during RKN parasitism, we performed reverse transcription quantitative real-time polymerase chain reaction (RT-qPCR) and virus-induced gene silencing (VIGS)-based functional verification of the candidate genes screened from the pseudotime trajectory analysis. In previous studies, transcriptomic profiling of *M. incognita*-induced galls and neighboring root cells demonstrated that *M. incognita* precisely manipulates the plant cell cycle, thereby elevating ploidy level at nematode feeding sites and accelerating mitotic activity in gall cells (Ozdemir et al. 2024). Moreover, unique transcription factors exhibit significant differential expression between gall tissues and adjacent root cells, further confirming the critical roles of cell cycle regulation and transcriptional reprogramming in gall cell differentiation (Ozdemir et al. 2024). Based on these findings, in this study, six candidate genes closely associated with cell cycle progression and gall development, including the B2-type cyclin-dependent kinase gene (*Solyc_MM_04G002940*), the cyclin-dependent kinase regulatory subunit 1 gene (*Solyc_MM_11G002008*), the G2/mitotic-specific cyclin-2 gene (*Solyc_MM_04G002896*), the cell division cycle 20.2 (cofactor of APC complex-like) gene (*Solyc_MM_06G002207*), the cell division control 20–1 gene (*Solyc_MM_03G001989*), and the transcription factor MYB3R-1-like gene (*Solyc_MM_08G001558*), were initially screened. All six genes had also been previously identified as being specifically expressed in giant cells during giant cell morphogenesis in a recent scRNA-seq study (Zadegan et al. 2025), validating their potential role in RKN-induced giant cell formation.

We further evaluated the six candidate genes according to their RT-qPCR expression patterns; specifically, genes that were significantly and stably upregulated in tomato root galls at the three key parasitic stages (3, 5, and 7 days post-*M. incognita* infection) were prioritized for subsequent VIGS functional validation. The RT-qPCR results revealed that four candidate genes, namely *Solyc_MM_04G002940*, *Solyc_MM_06G002207*, *Solyc_MM_03G001989*, and *Solyc_MM_08G001558*, were consistently upregulated at all three critical infection time points, indicating their close association with *M. incognita* parasitism and gall cell development (Fig. S22).

**Fig. 8:**
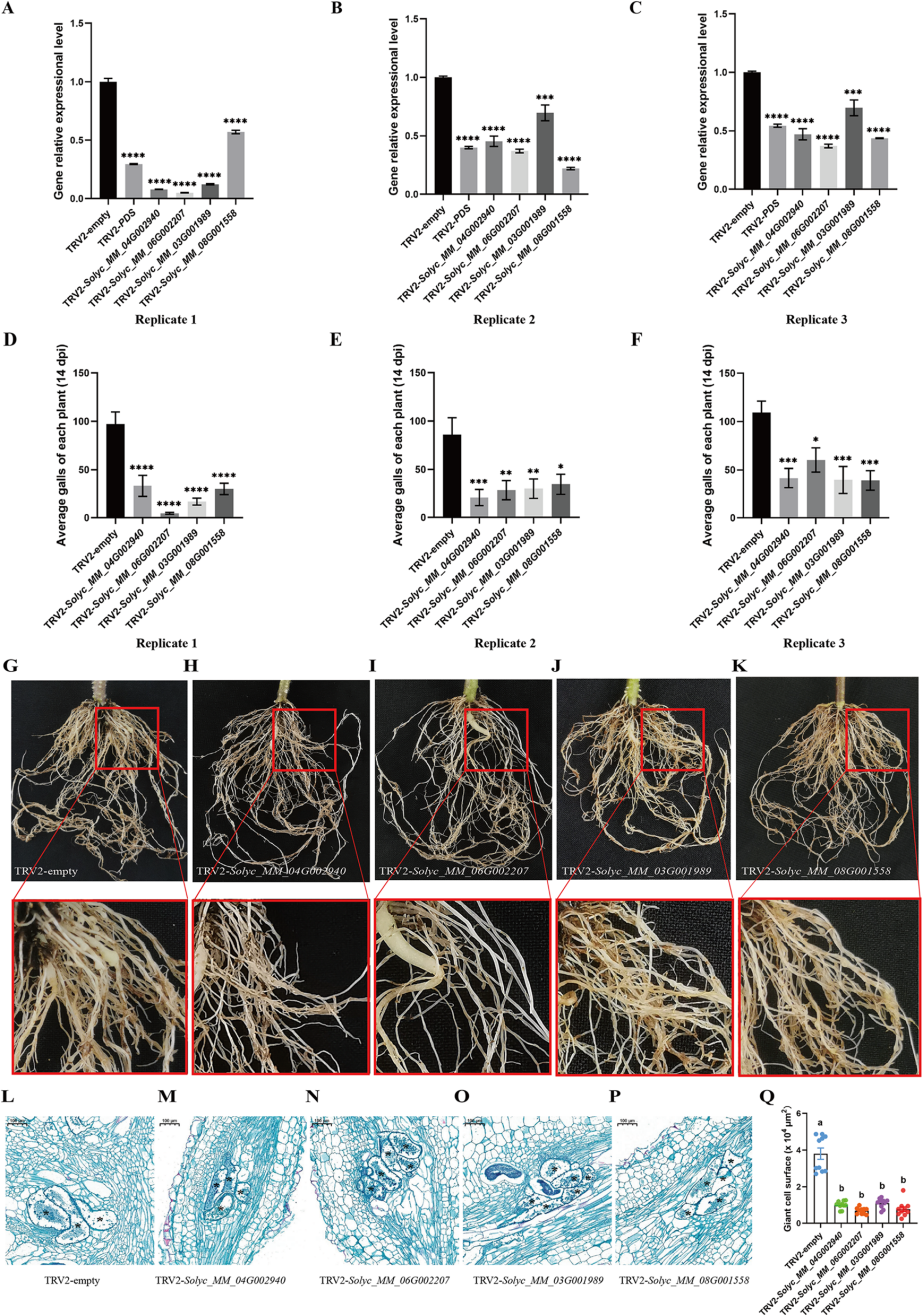
Functional verification of genes associated with the formation of giant cells. **A**-**C** RT-qPCR was performed to determine the relative expression levels of six selected candidate genes in the roots of *M. incognita*-infected tomato plants at 14 days post-infiltration (dpi). The tomato *ubiquitin* (*UBI*) gene was used for normalization. The bar chart shows the mean values ± SEM. The error bars represent SEM (*** *P* < 0.001, **** *P* < 0.0001). SEM, standard error of the mean. The data were analyzed with one-way ANOVA followed by Dunnett’s multiple comparisons test. **A**, **B**, and **C** represent three separate biological replicates. **D**-**F** Average number of galls of three independent biological replicates of VIGS-treated plants (> 10 plants) for the four genes at 14 days post-inoculation (dpi). The bar chart shows the mean values ± SEM. The error bars represent SEM (* *P* < 0.05, ** *P* < 0.01, *** *P* < 0.001, **** *P* < 0.0001). The data were analyzed with one-way ANOVA followed by Dunnett’s multiple comparisons test. **D**, **E**, and **F** represent three separate biological replicates. **G**-**K** Root phenotypes of tomato plants in which the four genes were silenced at 14 days post-inoculation (dpi). **L**-**P** Safranin O-Fast Green staining of gall sections of VIGS-treated plants at 14 days post-inoculation by *M. incognita*. Notably, when these genes were silenced, the giant cells in *Meloidogyne incognita*-induced galls were smaller than those in the control group. Asterisks represent giant cells. Scale bars, 100 μm. **Q** Giant cell areas of *M. incognita*-induced gall sections of VIGS-treated plants. Compared with the control treatment, gene silencing significantly reduced the size of the giant cells. The data are presented as the mean surface area ± standard error (SE) (n = 10). Different letters denote significant differences (*P* < 0.0001, one-way ANOVA)

These four genes were therefore selected for subsequent VIGS functional verification to further elucidate their biological roles in regulating giant cell formation. The silencing efficiency for these four genes on 14 dpi revealed that they were effectively knocked down (Fig. 8A–C). Compared with those of the control plants, the gall numbers in the gene-silenced tomato plants were significantly reduced 14 days post-inoculation with *M. incognita*, with average reduction rates of 68%, 69%, 70%, and 64%, respectively (Fig. 8D–K). Considering the temporal dynamics of giant cell development, our investigation was conducted at a relatively early stage, and no significant variation in the development patterns of the tomato root system was observed (Fig. 8G–K). To present a clearer picture of how these four genes influence giant cell formation, we stained gall sections from VIGS-treated plants at 14 days post-inoculation with *M. incognita* using Safranin O-Fast Green. The results showed that the silencing of these four genes separately led to significantly smaller giant cells than those in the control group (Fig. 8L–Q). It is worth mentioning that *Solyc_MM_04G002940* encodes a cyclin-dependent kinase crucial for G1/S and G2/M phase transitions, guaranteeing normal cell cycle progression; *Solyc_MM_06G002207* and *Solyc_MM_03G001989* are cell division cycle-associated protein-encoding genes. These types of genes are known to govern orderly cell division cycle progression and ensure accurate initiation, progression, and transition across all stages (from G1 phase, S phase, and G2 phase to M phase). In tobacco, NtmybA1 and NtmybA2 bind to mitosis-specific activator (MSA) elements; two structurally related genes—*MYB3R1* and *MYB3R4*, which encode homologs of NtmybA1 and NtmybA2 in *Arabidopsis thaliana*—play partially redundant roles in positively regulating cytokinesis (Haga et al. 2007). As we found that *Solyc_MM_08G001558* encodes a MYB3R-1-like transcription factor in our research, we speculate that this gene is also associated with giant cell formation, representing, for the first time, the involvement of a tomato MYB3R transcription factor in RKN-induced giant cell formation. We speculate that these genes may interfere with the normal cell cycle or gene regulation, thereby participating in abnormal cell division and the formation of multinucleated giant cells during RKN infection (a working model is depicted in Fig. S23).

## Discussion

Deciphering the pathogenic mechanisms underlying giant cell initiation, developmental progression, and RKN feeding site establishment remains a critical challenge (Favery et al. 2016; Olmo et al. 2020). In this study, we analyzed spatial transcriptomic datasets from *M. incognita*-infected Moneymaker tomato root gall tissue sections at 3, 5, and 7 days post-inoculation using the BMKMANU S1000 platform. This work represents the first application of spatial transcriptomic sequencing for investigating the mechanism underlying RKN-induced giant cell formation in tomato roots, leveraging its advantage of retaining the spatial localization information of gene expression compared to snRNA-seq, whereas scRNA-seq is unsuitable because of the large size of giant cells (Giacomello 2021; Li and Wang 2021). On the basis of spatial expression patterns of spots, combined with tomato-specific markers derived from homology alignment with sequences of previously reported species (Lv et al. 2024), we classified and identified various cell types, including cortex, meristem, phloem, stele, and xylem, with their spatial positions vividly visualized. This study provides valuable references for spatial cell type identification in heterogeneous tissues.

RKNs invade host roots via the elongation zone, migrate to the root meristem, and establish parasitism in the vascular system, inducing differentiation of several vascular cells into giant cells (Rodiuc et al. 2014; Wyss et al. 1992). The establishment of the RKN feeding site, which is surrounded by 7–8 giant cells, alongside asymmetric division in adjacent cells, relies on the regulation of gene expression in RKN-infected root cells (Cabral et al. 2020). Previous studies have used multiple techniques to identify numerous DEGs linked to cell cycle activation, hormone signaling, defense response, and cell wall modification during feeding site formation (Caillaud et al. 2008a; Gheysen and Fenoll 2002; Quentin et al. 2013). In this study, tomato homologs of *A. thaliana* auxin-responsive factor genes were used as molecular markers, assisting in the identification of giant cell-specific clusters—in which these genes were highly expressed—across all three samples. Similar results emerged for the tomato *ADF2* homolog. Tomato homologs of *Atcdc2a* and *AtCKS2* exhibited high expression in several clusters of the 3 dpi and 7 dpi samples. Two homologs of the mitotic cyclin gene *Atcyc1* were highly expressed in two clusters of the Day 3 sample. Additionally, homologs of the root apical meristem regulators *SHR* (*Solyc_MM_02G003216*), *CCS52* (*Solyc_MM_06G000850*), and *MAP65-3* (*Solyc_MM_03G000205*) were highly expressed in clusters 17 and 13 of the Day 3 sample. Using this “homologous marker gene” approach, we successfully identified giant cell-specific clusters (xylem, stele, and meristem); notably, the meristem tissue cell type, which was reported to possess transient pluripotency for gall organogenesis (Olmo et al. 2020), was uncovered and supplemented in our research, advancing the results from the recent report by Zadegan et al. (2025). GO enrichment analysis of upregulated DEGs in giant cell clusters and single-gene spatial visualization and gene set scoring of marker genes further validated the reliability of our cluster annotations. Analysis of the cell–cell communication between giant cells and adjacent cells revealed the dynamic changes in intercellular cross-talk throughout the progression of RKN infection. DEG analysis between giant cell and normal cell clusters revealed novel marker genes that showed strong correspondence with giant cells, which were subsequently verified via in situ RNA hybridization. In summary, in this study, we systematically characterized giant cell-specific clusters and discovered novel marker genes, providing critical new avenues for research on RKN parasitism.

The development of normal vascular bundle cells into giant cells is critical for the formation of RKNs’ feeding sites. In our study, pseudotime analysis (Zhao et al. 2025) was performed to explore vascular bundle cell differentiation trajectories, and multiple genes involved in cell fate branches were identified, providing a new perspective for understanding the mechanisms underlying giant cell formation. We found that vascular bundle cells differentiated along two distinct paths: one leading to the formation of normal cells and the other to giant cells, with numerous DEGs that may be involved in giant cell formation identified (Table S14). The eukaryotic cell cycle is a highly regulated process that is essential for proper DNA replication and successful cytokinesis. RKNs induce giant cell formation by promoting mitotic cycles without complete cytokinesis, wherein transcriptional activation of cyclin-dependent kinases and mitotic cyclins is closely associated with the early stage of giant cell development (Niebel et al. 1996). Analysis of the 3 dpi sample revealed cell cycle regulation-related DEGs (such as cyclin-dependent kinase genes, G2/mitotic-specific cyclin genes, the *CCS52B* gene, cell division cycle-associated protein genes, and the MYB3R transcription factor gene), highlighting their key role in giant cell development in the initial stage. As nutrient reservoirs for RKNs, giant cells rely on extensive material transport to support their formation (Rodiuc et al. 2014). The genes that encode early nodulin-like protein, tubulin alpha chain-like protein, and aquaporin were identified in samples at 3 dpi and 7 dpi, indicating that they may be involved in active material transport throughout giant cell development (Ding et al. 2019; Ntui et al. 2024; Pastuglia and Bouchez 2007). During the transition phase (5 dpi) and near-maturity phase (7 dpi) of giant cell formation, we identified cell division-, growth regulation-related transcription factor-, and translation initiation and elongation factor-encoding genes, suggesting that the sustained proliferation of giant cells post-initiation may correlate with the regulation of these genes (Feng et al. 2007; Hossain et al. 2012; Sozzani et al. 2010; Thompson et al. 2004; Zhao et al. 2024). Additionally, two HMG protein-encoding genes, several histone-related protein-encoding genes, and an AUX/IAA protein-encoding gene were identified in the Day 7 sample, reinforcing their associations with DNA replication/processing and hormonal regulation (Bustin 1999; Foroozani et al. 2022; Olmo et al. 2020). Notably, many cell cycle-related genes identified in the Day 3 sample overlapped with recent single-cell nuclear sequencing-identified giant cell-specific genes (Zadegan et al. 2025), indicating their prominent role in regulating giant cell formation.

To further elucidate the roles of the potential giant cell formation-related genes identified via pseudotime analysis, three genes potentially associated with cell division cycle regulation and one gene that encodes an MYB3R transcription factor that might regulate cytokinesis—which are upregulated in *M. incognita*-infected tomato root gall—were functionally characterized through VIGS-mediated gene silencing in tomato plants. The results exhibited markedly fewer galls and smaller giant cells in VIGS-silenced tomato roots at 14 days post-inoculation, confirming the essential contributions of the four candidate genes to the development of the RKN feeding site. These genes also possess prominent biocontrol potential for RKN management, owing to their specific activation during RKN infection and gall formation. Targeted suppression of these cell cycle and cytokinesis regulatory genes could effectively impair feeding site maturation and limit the capability of RKNs for parasitic colonization and nutrient acquisition. These genes may therefore serve as potential candidate knockdown molecular targets for future improvements of nematode resistance strategies in the molecular breeding of tomato. Our research established the first spatial transcriptional profile of RKN-infected tomato roots, revealing multiple key genes linked to giant cell formation, which possess significant strategic value for understanding the pathogenic mechanisms of RKNs and provide precise targets for RKN management.

## Materials and methods

### Plant and nematode materials

Tomato plants (*Solanum lycopersicum* var. “Moneymaker”) were grown in pots with nutrient soil and vermiculite (3:1) and cultivated under a 16-h light/8-h dark photoperiod. *M. incognita* was propagated on *Ipomoea aquatica Forsk* under the same conditions. Egg masses were collected from galled roots and hatched in water at 25 ℃ to obtain pre-parasitic second-stage juveniles (pre-J2s) of *M. incognita*.

### Spatial transcriptomic tissue preparation and frozen section embedding

Moneymaker tomato plants were inoculated with 5000 pre-J2s of *M. incognita* through three holes near the root zone at the 3–5 leaf stage (Lizardo et al. 2022)*.* The root galls from at least five individual plants were harvested at days 3, 5, and 7 post-inoculation (dpi), respectively. Collected galls were rinsed with sterile water and blotted dry with sterile paper. The tissues were minced into small fragments (~6.8 mm^2^) and immediately snap-frozen in liquid nitrogen-cooled prechilled isopentane. Subsequently, all tissue fragments were embedded in optimal cutting temperature (OCT) compound (SAKURA, Cat#: 4583) and stored at −80 °C. Four gall tissues gathered at each of the three time points were embedded separately for subsequent transcriptomic profiling.

### Tissue fixation, staining, and imaging

The samples were frozen-sectioned at a thickness of 15 μm. Following fixation, the sections were stained with toluidine blue, and the target structure was identified based on the characteristics of the giant cell organization. Once the targets appeared, the second or third slice was placed on chilled BMKMANU S1000 Gene Expression Slides (each with eight capture areas of 6.8 × 6.8 mm, one area containing 2,000,000 spots with barcoded primers and a diameter of 2.5 μm) for standard toluidine blue staining and subsequent spatial transcriptomic analysis, while the next four to six slices were systematically placed on tissue optimization slides for tissue optimization. The Spatial Transcriptomics (ST) protocol was optimized using the BMKMANU S1000 Tissue Optimization Kit (BMKMANU, ST03003), in accordance with established guidelines (Lyu et al. 2024). Images were captured at 40 × magnification with a Pannoramic MIDI microscope.

### Library construction and sequencing on an Illumina NovaSeq 6000 platform

cDNA sequencing libraries were constructed via reverse transcription (RT) and adaptor ligation and then processed according to the manufacturer’s instructions using a Library Construction Kit (BMKMANU, ST03002-34). The completed libraries were purified and quantified prior to sequencing on the Illumina NovaSeq 6000 platform by Biomarker Technologies Corporation (Beijing, China).

### BMKMANU S1000 spatial gene expression analysis

Raw data were obtained by converting the raw image files into sequenced reads using Illumina BCL Convert software (Version 00.000.000.4.2.7). The clean data were subjected to further analysis after quality assessment. The Moneymaker tomato reference genome was referenced as previously reported (Zhou et al. 2022). A spot-gene expression matrix was generated by aligning the data to the reference genome using STAR (v.2.5.1b) (Dobin et al. 2013). Tissue detection and spatial barcode/UMI statistics were calculated using BSTMatrix (v2.3.q) (http://www.bmkmanu.com/portfolio/tools) to obtain the quantified gene expression in the spots.

Data analysis was performed using the Seurat R package (v4.0.1) (Satija et al. 2015); the sctransform (SCT) algorithm in the Seurat package was used to standardize the data. Afterward, the VST algorithm was used to identify high-variance genes (HVGs) according to the default parameters. Subsequently, the HVGs were utilized for PCA/UMAP (Becht et al. 2018) reduction. The resolution level of 2.0 was employed to divide spatial spots into different regional clusters. Visualization was achieved using the ggplot2 R package (Wickham 2016). After spot clustering, the Wilcoxon rank-sum test was performed to identify DEGs among distinct clusters. Genes with a fold change (FC) ≥ 1.5 and a p-value < 0.05 were defined as cluster-specific marker genes and DEGs. Gene Ontology (GO) enrichment analysis of DEGs that were upregulated among the giant cell clusters was performed using the clusterProfiler R package (4.4.4) with default parameters. Significantly enriched GO terms were identified based on a threshold of p-value < 0.05.

To ensure the comparability of the spatial transcriptomic data at the three time points, we adopted a uniform standardized pretreatment and normalization process for all samples, including uniform background correction and feature count normalization.

### Cell–cell communication analysis

A total of 1,751 ligand-receptor pairs from tomato were downloaded from the PlantPhoneDB database (https://jasonxu.shinyapps.io/PlantPhoneDB/) (Xu et al. 2022). Tomato homologs of these genes were identified via RBH and BLAST analysis. Spots of giant cells and their three adjacent neighbors were extracted, with spots belonging to clusters annotated as “Unknown” excluded. Cell–cell communication analysis was conducted separately for the three samples using the PlantPhoneDB R package (v1.0.0) with the following parameters: min.pct = 0.1, iterations = 100, and method = “Average”. Finally, the communication results across the different samples were compared.

### Spatial transcriptomic trajectory analysis

The cell differentiation trajectory in pseudotime was analyzed using the Monocle 2 package (version 2.18.0; Monocle, USA) (Qiu et al. 2017; Trapnell et al. 2014). The “detectGenes” and “subset” functions were set to “min_expr = 0.2” and “num_cells_expressed ≥ 100”, respectively. For dimension reduction, the DDRTree method was used in the “reduceDimension” function with “max_components = 2”. The “differentialGeneTest” function (parameters “cores = 4” and “q-value < 1e-4”) identified specific genes across multiple cell subtypes, and “orderCells” arranged cells by pseudotime progression. The cell differentiation trajectories and states were visualized in two-dimensional space via “plot_cell_trajectory”. BEAM analysis of characteristic gene expression clustering with pseudotime values was performed using “plot_genes_branched_heatmap” (num_clusters = 6, cores = 8).

### RNA in situ hybridization and VIGS verification

RNA in situ hybridization was performed to investigate the spatial expression of novel giant cell marker genes. Samples were rinsed with phosphate-buffered saline (PBS), fixed in plant in situ hybridization fixative (Servicebio Technology Co., Ltd., Wuhan, China) for over 12 h, and vacuum-extracted. The tissue blocks were sectioned to 3 mm, dehydrated in a graded series of alcohols, cleared with xylene, embedded in paraffin, and sliced to 6 μm using a pathology slicer (Leica, Shanghai, China). The slices were then incubated at 62 ℃ for 2 h. Probes with the 5’ T7 promoter sequence “ggcTAATACGACTCACTATAGGG” were amplified; antisense probes were synthesized via T7 RNA polymerase (MAXIscript™ T7 Transcription Kit, Thermo) and labeled with digoxigenin (DIG RNA Labeling mixture, Roche) (primers/probes in Table S15). The sections were dewaxed, cleaned with DEPC water, digested with 20 μg/mL protease K (Servicebio) at 37 ℃, hybridized, and incubated with AP-labeled mouse anti-digoxigenin antibody (anti-DIG-AP, Jackson, America) at 37 ℃ for 40 min. Signals were detected with BCIP/NBT stock solution (Boster, Wuhan, China), and images were captured with a Pannoramic^®^250 Flash III microscope slide scanner (3DHISTECH Ltd, Budapest, Hungary).

RT-qPCR was performed to validate candidate giant cell formation-associated genes uncovered from pseudotime trajectory analysis and upregulated in *M. incognita*-infected tomato root. VIGS was used to validate the functions of these genes in giant cell formation following previously described methods (Jiang et al. 2023) (primers in Table S16; tomato *ubiquitin* (*UBI*) as reference). Two weeks post-infiltration, VIGS-treated tomato plants were inoculated with 600 M*. incognita* pre-J2s, and the root galls were evaluated 14 days post-inoculation (3 independent biological replicates; 10–15 plants/treatment). At least 10 galls for each VIGS-treated line were embedded in paraffin, sectioned, and stained with Safranin O-Fast Green (Servicebio, G1031). The sections were then scanned (Pannoramic^®^250 Flash III) and imaged (Saiviewer-1.0.9, Servicebio). The areas of giant cells (≥ 10/line) were measured with Image-Pro Plus 6.0 (Media Cybernetics), and the data were statistically analyzed via one-way ANOVA followed by Dunnett’s multiple comparisons test (GraphPad Prism 8.0).

## Supplementary Information

Supplementary Material 1: Supplementary Fig. S1-S10. Fig. S1. Toluidine blue staining of gall tissue sections collected at three post-infection time points for spatially resolved transcriptomic analysis. Fig. S2. Spatial distribution map of tissue section spots and numbers of unique molecular identifiers (UMIs)/genes in the spots. Fig. S3. Spatial distribution of clusters in the Day 3 sample. Fig. S4. Spatial distribution of clusters in the Day 5 sample. Fig. S5. Spatial distribution of clusters in the Day 7 sample. Fig. S6. Violin plots of the top 10 feature genes in each cluster of the Day 3 sample. Fig. S7. Violin plots of the top 10 feature genes in each cluster of the Day 5 sample. Fig. S8. Violin plots of the top 10 feature genes in clusters 0–15 of the Day 7 sample. Fig. S9. Violin plots of the top 10 feature genes in clusters 16–24 of the Day 7 sample. Fig. S10. Violin plots of the number of genes detected in the cluster spots.

Supplementary Material 2: Supplementary Fig. S11-S23. Fig. S11. Spatial expression distribution maps of marker genes for identifying giant cells. Fig. S12. GO enrichment analysis of upregulated differentially expressed genes (DEGs) in the giant cell-specific clusters in the three samples. Fig. S13. Spatial expression distribution of each type of representative tissue marker (gene by gene) in the Day 3 sample. Fig. S14. Spatial expression distribution of each type of representative tissue marker (gene by gene) in the Day 5 sample. Fig. S15. Spatial expression distribution of each type of representative tissue marker (gene by gene) in the Day 7 sample. Fig. S16. Spatial region gene set scoring of the entire set of tissue markers of the Day 3 sample. Fig. S17. Spatial region gene set scoring of the entire set of tissue markers of the Day 5 sample. Fig. S18. Spatial region gene set scoring of the entire set of tissue markers of the Day 7 sample. Fig. S19. Spatial expression distribution maps of potential giant cell marker genes in the giant cell-specific clusters of the three samples. Fig. S20. Venn diagram and spatial region gene set scoring of the overlapping giant cell-specific (upregulated) genes between our research and an snRNA-seq study. Fig. S21. The expression dynamics of genes with essential functions at branch points in the differentiation of normal vascular bundle cells to giant cells. Fig. S22. Relative gene expression levels of six selected candidate genes in the root galls of *M. incognita*-infected tomato plants. Fig. S23. Working model illustrating the speculated molecular mechanism through which these four genes participate in giant cell formation.

Supplementary Material 3: Supplementary Table S1-S16. Table S1. Statistics of sequencing data analysis sequences. Table S2. Supspot-level resolution description. Table S3. Statistics information for supspots in tissue sections. Table S4. Average expression of genes for all identified clusters. Table S5. The expression levels of the top 10 feature genes in each cluster of the Day 3 sample. Table S6. The expression levels of the top 10 feature genes in each cluster of the Day 5 sample. Table S7. The expression levels of the top 10 feature genes in each cluster of the Day 7 sample. Table S8. Cell type annotation for the clusters. Table S9. Marker genes used for the giant cell-specific clusters. Table S10. GO enrichment analysis of upregulated differentially expressed genes (DEGs) in the giant cell-specific clusters of the three samples. Table S11. Cell–cell communication between giant cells and their surrounding cells. Table S12. Differentially expressed genes in giant cell-specific clusters and the identified novel marker genes. Table S13. Functional annotation of differentially expressed genes associated with giant cell differentiation in pseudotime trajectory analysis and key potential genes discovered therein. Table S14. Comparison of DEGs in our pseudotime trajectory analysis and the reported 772 genes specifically expressed in giant cells in recent single-nucleus RNA sequencing studies. Table S15. Primers and probes used for RNA in situ hybridization. Table S16. Primers used for the VIGS experiments.

## Data Availability

All spatial transcriptomic data in this paper are available at NCBI Gene Expression Omnibus (GEO) with Bioproject accession number PRJNA1196910 and GEO accession number GSE284052. When the data is in private status, reviewers can access it using the accession number and GEO reviewer token: ulynuaqkljwtrof. The data will be immediately released upon acceptance of the article for publication.

## Abbreviations

*ADF2*: *actin-depolymerizing factor 2*
*A. thaliana*: *Arabidopsis thaliana*
*ARF5*: *AUXIN-RESPONSIVE-FACTOR-5*
*CCS52s*: cell cycle switch gene encoding a 52 kDa protein
CDK: cyclin-dependent kinase
DEGs: Differentially expressed genes
*DEL1*: *DP-E2F-like 1*
HMG: high mobility group
HVGs: high-variance genes
*KRP6*: *Kip-related protein 6*
*M. incognita*: *Meloidogyne incognita*
MAP65-3: *Microtubule-Associated Protein 65-3*
OCT: optimal cutting temperature
PBS: Phosphate-Buffered Saline
pre-J2s: pre-parasitic second-stage juveniles
RT: reverse transcription
RT-qPCR: reverse transcription quantitative real-time polymerase chain reaction
RKNs: Root-knot nematodes
SCT: sctransform
J2s: second-stage juvenile
*SHR*: *SHORT-ROOT*
scRNA-seq: single-cell RNA-seq
snRNA-seq: single-nucleus RNA-seq
Stereo-seq: SpaTial Enhanced REsolution Omics-sequencing
ST: Spatial Transcriptomics
UMAP: Uniform manifold approximation and projection
*UBI*: *Ubiquitin*
UMIs: Unique Molecular Identifiers
VIGS: virus-induced gene silencing
