# Supplementary material for: A spatiotemporal atlas of giant-cell formation during Meloidogyne incognita infection in tomato roots
Source: Mol Hortic. 2026 Aug 11;6:66. doi: 10.1186/s43897-026-00272-5 (PMC13459382; doi:10.1186/s43897-026-00272-5)
Supplement: Supplementary file 1 [file 43897_2026_272_MOESM1_ESM.docx]

**Fig. S1** Toluidine blue staining of gall tissue sections collected at three post-infection time points for spatially resolved transcriptomic analysis. (A) Root tissue section of Moneymaker tomato on the third day after inoculation with *M. incognita*. (B) Root tissue section of Moneymaker tomato on the fifth day after inoculation with *M. incognita*. (C) Root tissue section of Moneymaker tomato on the seventh day after inoculation with *M. incognita.*

**Fig. S2** Spatial distribution map of tissue section spots and numbers of unique molecular identifiers (UMIs)/genes in the spots. (A), (D), (G) Spatial distribution map of tissue section spots of samples on the third, fifth, and seventh day, respectively. (B), (E), (H) Number of genes in tissue section spots of samples on the third, fifth, and seventh day, respectively. (C), (F), (I) Numbers of UMIs in tissue section spots of samples on the third, fifth, and seventh day, respectively. Each point represents a spot in a spatial location. The colors in the bar chart represent the relative abundance of UMIs or gene numbers in the spots, and the scale value indicates the number of UMIs or genes.

**Fig. S3** Spatial distribution of clusters in the Day 3 sample.

**Fig. S4** Spatial distribution of clusters in the Day 5 sample.

**Fig. S5** Spatial distribution of clusters in the Day 7 sample.

**Fig. S6** Violin plots of the top 10 feature genes in each cluster of the Day 3 sample. The horizontal coordinate represents the gene expression value, and the vertical coordinate represents the spot cluster. The expression levels of feature genes in different clusters can be intuitively seen through this figure.

**Fig. S7** Violin plots of the top 10 feature genes in each cluster of the Day 5 sample. The horizontal coordinate represents the gene expression value, and the vertical coordinate represents the spot cluster. The expression levels of feature genes in different clusters can be intuitively seen through this figure.

**Fig. S8** Violin plots of the top 10 feature genes in clusters 0-15 of the Day 7 sample. The horizontal coordinate represents the gene expression value, and the vertical coordinate represents the spot cluster. The expression levels of feature genes in different clusters can be intuitively seen through this figure.

**Fig. S9** Violin plots of the top 10 feature genes in clusters 16-24 of the Day 7 sample. The horizontal coordinate represents the gene expression value, and the vertical coordinate represents the spot cluster. The expression levels of feature genes in different clusters can be intuitively seen through this figure.

**Fig. S10** Violin plots of the number of genes detected in the cluster spots. The horizontal axis represents clusters, and the vertical axis represents the number of genes.
