# Supplementary material for: A spatiotemporal atlas of giant-cell formation during Meloidogyne incognita infection in tomato roots
Source: Mol Hortic. 2026 Aug 11;6:66. doi: 10.1186/s43897-026-00272-5 (PMC13459382; doi:10.1186/s43897-026-00272-5)
Supplement: Supplementary file 2 [file 43897_2026_272_MOESM2_ESM.docx]

**Fig. S11** Spatial expression distribution maps of marker genes for identifying giant cells. Each point represents a spatial spot. The colors represent the expression level of the genes; red indicates a high expression level, and blue indicates a low expression level.

**Fig. S12** GO enrichment analysis of upregulated differentially expressed genes (DEGs) in the giant cell-specific clusters in the three samples. Representative and significant GO terms (p-value <0.05) are shown. (A)-(C) indicate the analysis of the third-day sample, the fifth-day sample, and the seventh-day sample, respectively.

**Fig. S13** Spatial expression distribution of each type of representative tissue marker (gene by gene) in the Day 3 sample. Each point represents a spatial spot. The colors represent the expression level of the genes; red indicates a high expression level, and blue indicates a low expression level.

**Fig. S14** Spatial expression distribution of each type of representative tissue marker (gene by gene) in the Day 5 sample. Each point represents a spatial spot. The colors represent the expression level of the genes; red indicates a high expression level, and blue indicates a low expression level.

**Fig. S15** Spatial expression distribution of each type of representative tissue marker (gene by gene) in the Day 7 sample. Each point represents a spatial spot. The colors represent the expression level of the genes; red indicates a high expression level, and blue indicates a low expression level.

**Fig. S16** Spatial region gene set scoring of the entire set of tissue markers of the Day 3 sample. Each point represents a spatial spot, and the colors indicate the size of the score.

**Fig. S17** Spatial region gene set scoring of the entire set of tissue markers of the Day 5 sample. Each point represents a spatial spot, and the colors indicate the size of the score.

**Fig. S18** Spatial region gene set scoring of the entire set of tissue markers of the Day 7 sample. Each point represents a spatial spot, and the colors indicate the size of the score.

**Fig. S19** Spatial expression distribution maps of potential giant cell marker genes in the giant cell-specific clusters of the three samples. Each point represents a spatial spot. The colors denote the gene expression levels; red indicates a high expression level, while blue indicates a low expression level.

**Fig. S20** Venn diagram and spatial region gene set scoring of the overlapping giant cell-specific (upregulated) genes between our research and an snRNA-seq study. (A)-(C) Venn diagram of the overlapping genes. (D)-(F) Spatial region gene set scoring of the overlapping genes. Each point represents a spatial spot, and the colors indicate the size of the score.

**Fig. S21** The expression dynamics of genes with essential functions at branch points in the differentiation of normal vascular bundle cells to giant cells. The horizontal coordinate represents the pseudo-time point, while the vertical coordinate represents the gene expression level. Each dot represents a spatial spot, and different colors denote the different cell type clusters. The dashed line and black solid line represent the gene expression dynamic trends of the giant cell precursor branch and normal cell precursor branch, respectively.

**Fig. S22** Relative gene expression levels of six selected candidate genes in the root galls of *M. incognita*-infected tomato plants. RT-qPCR was performed to detect the gene relative expression level of the candidate genes in the root galls of *M. incognita*-infected tomato plants at the third (A), fifth (B), and seventh (C) days. The healthy tomato roots, which were not inoculated with RKNs, served as the control. The ubiquitin (UBI) gene was used for normalization. The bar chart represents the mean values ± SEM. The error bars represent SEMs (*** *P* < 0.001, **** *P* < 0.0001). SEM means standard error of the mean. Data were analyzed by one-way ANOVA followed by Dunnett’s multiple comparisons test.

**Fig. S23** Working model illustrating the speculated molecular mechanism through which these four genes participate in giant cell formation.
